# A Stress Response Monitoring Lipoprotein Trafficking to the Outer Membrane

**DOI:** 10.1128/mBio.00618-19

**Published:** 2019-05-28

**Authors:** Kerrie L. May, Kelly M. Lehman, Angela M. Mitchell, Marcin Grabowicz

**Affiliations:** a Emory Antibiotic Resistance Center, Emory University School of Medicine, Atlanta, Georgia, USA; b Department of Microbiology & Immunology, Emory University School of Medicine, Atlanta, Georgia, USA; c Division of Infectious Diseases, Department of Medicine, Emory University School of Medicine, Atlanta, Georgia, USA; d Microbiology and Molecular Genetics Program, Graduate Division of Biological and Biomedical Sciences, Emory University School of Medicine, Atlanta, Georgia, USA; e Department of Molecular Biology, Princeton University, Princeton, New Jersey, USA; Vanderbilt University Medical Center; Washington University School of Medicine

**Keywords:** Cpx response, Lol pathway, NlpE, copper, envelope stress response, lipoproteins, outer membrane

## Abstract

Gram-negative bacteria produce lipid-anchored lipoproteins that are trafficked to their outer membrane (OM). These lipoproteins are essential components in each of the molecular machines that build the OM, including the Bam machine that assembles β-barrel proteins and the Lpt pathway that transports lipopolysaccharide. Stress responses are known to monitor Bam and Lpt function, yet no stress system has been found that oversees the fundamental process of lipoprotein trafficking. We used genetic and chemical biology approaches to induce several different lipoprotein trafficking stresses in Escherichia coli. Our results identified the Cpx two-component system as a stress response for monitoring trafficking. Cpx is activated by trafficking defects and is required to protect the cell against the consequence of the resulting stress. The OM-targeted lipoprotein NlpE acts as a sensor that allows Cpx to gauge trafficking efficiency. We reveal that NlpE signals to Cpx while it is transiting the inner membrane (IM) *en route* to the OM and that only a small highly conserved N-terminal domain is required for signaling. We propose that defective trafficking causes NlpE to accumulate in the IM, activating Cpx to mount a transcriptional response that protects cells. Furthermore, we reconcile this new role of NlpE in signaling trafficking defects with its previously proposed role in sensing copper (Cu) stress by demonstrating that Cu impairs acylation of lipoproteins and, consequently, their trafficking to the OM.

## Abstract (precis)

The outer membrane built by Gram-negative bacteria such as Escherichia coli forms a barrier that prevents antibiotics from entering the cell, limiting clinical options at a time of prevalent antibiotic resistance. Stress responses ensure that barrier integrity is continuously maintained. We have identified the Cpx signal transduction system as a stress response that monitors the trafficking of lipid-anchored lipoproteins to the outer membrane. These lipoproteins are needed by every machine that builds the outer membrane. Cpx monitors just one lipoprotein, NlpE, to detect the efficiency of lipoprotein trafficking in the cell. NlpE and Cpx were previously shown to play a role in resistance to copper. We show that copper blocks lipoprotein trafficking, reconciling old and new observations. Copper is an important element in innate immunity against pathogens, and our findings suggest that NlpE and Cpx help E. coli survive the assault of copper on a key outer membrane assembly pathway.

## INTRODUCTION

The outer membrane (OM) is an essential organelle for Gram-negative bacteria such as Escherichia coli (1, 2). The OM is an asymmetrical lipid bilayer consisting of phospholipids in the inner leaflet and lipopolysaccharide (LPS) in the surface-exposed outer leaflet (3). Two types of proteins reside in the OM: (i) β-barrel outer membrane proteins (OMPs) form transmembrane channels, and (ii) lipoproteins, a family of acylated proteins, are anchored in the OM bilayer and fulfil diverse functions (1). All of the OM components are synthesized in the cytosol or at the inner membrane (IM). Each of these highly hydrophobic molecules must be transported across the unfavorable aqueous periplasmic environment to the OM and assembled into the bilayer in a compartment lacking sources of chemical energy such as ATP (2, 4, 5).

Several OM assembly machines have been identified. LPS is transported and assembled via the Lpt pathway (4). Nascent secreted OMPs in their unfolded form are transported by periplasmic chaperones to the Bam machine that folds and inserts them into the OM (2, 6, 7). For OM-targeted lipoproteins, the Lol pathway is the major trafficking route that brings lipoproteins from the IM, where they are acylated, to the OM (5, 8). All the complex OM assembly processes must remain highly choreographed so that OM integrity can be continuously maintained. Accordingly, several stress responses have been discovered that underpin OM biogenesis by monitoring the fidelity of assembly processes and responding when defects arise to protect the cell (9–12). LPS defects at the OM are primarily sensed by the Rcs stress response which upregulates production of exopolysaccharides that protect the OM (9, 13). OMP biogenesis is monitored primarily by the σ^E^ response that functions to balance rates of new OMP synthesis with rates of OMP assembly into the OM, thereby protecting the cell against toxic accumulation of OMPs in the periplasm (10, 14–16). Both these responses are also induced by other stresses: Rcs is also activated by *bam* mutations (17, 18), and σ^E^ also responds to defects in LPS structure or transport (19).

Lipoproteins are key players in OM assembly. Each of the OM assembly machines requires at least one essential OM lipoprotein for function (5). In the Bam machine, BamD is an essential OM lipoprotein, while BamBCE are accessory lipoproteins that are collectively essential (20, 21). The OM lipoprotein LptE is essential for the Lpt pathway (22). Even in the Lol lipoprotein trafficking pathway, LolB is an essential OM lipoprotein (23).

**FIG 1:**
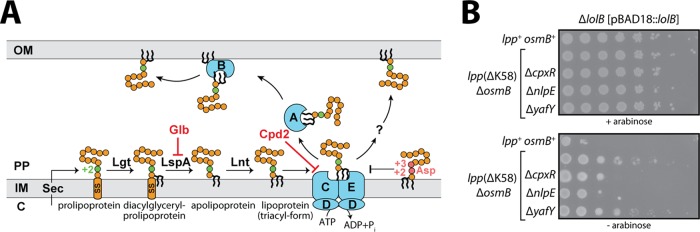
NlpE and Cpx are required for tolerance of LolB depletion. (A) Overview of lipoprotein biogenesis and trafficking. Lipoproteins are secreted via the Sec translocon and are acylated at Cys^+1^ in the IM. Mature triacylated lipoproteins that are targeted for the OM enter LolCDE for extraction from the IM. LolA and LolB are part of an efficient trafficking pathway that is essential in wild-type cells. An alternate LolAB-independent pathway can also traffic lipoproteins but is insufficient in wild-type cells. Asp residues at +2 and +3 amino acids cause IM retention of lipoproteins. The targets of Glb and Cpd2 inhibitors are shown. (B) Strains tested for tolerance to LolB depletion. Expression of LolB was repressed by culturing in the absence of l-arabinose. Ten-fold serial dilutions of cultures are shown.

Once translated, lipoproteins are translocated from the cytosol and then modified with an *S*-diacylglyceryl moiety at an invariant Cys residue by the enzyme Lgt (Fig. 1A) (24, 25). The resultant diacyl form lipoprotein becomes a substrate for signal peptidase II (Lsp) that cleaves adjacent to the lipidated Cys, liberating the amino group of this residue (Fig. 1A) (26, 27). The enzyme Lnt then catalyzes an *N*-acylation of the Cys (Fig. 1A) (28). In E. coli, only a minority of lipoproteins are retained in the IM; these have Asp^+2^ residues that prevent their trafficking (29, 30). The OM-targeted lipoproteins are first extracted from the IM by the LolCDE transporter (Fig. 1A). Only mature triacyl form lipoproteins can interact with the LolCDE complex (31). LolCDE use ATP hydrolysis to power the extraction of lipoproteins from the IM bilayer (32, 33). There are at least two subsequent trafficking routes that lipoproteins take to reach the OM. LolA and LolB provide a highly efficient route in wild-type cells: the periplasmic chaperone protein LolA receives lipoproteins from LolCDE and shuttles them across the periplasm (Fig. 1A) (34, 35), and at the OM, LolB receives lipoproteins from LolA and anchors them in the bilayer (Fig. 1A) (23, 36, 37). Recent work identified a mutant E. coli strain that tolerates deletion of both *lolA* and *lolB*, revealing that at least one additional trafficking route must exist that can support viability (38).

Since lipoproteins are requisite components in each of the essential OM assembly machines and these machines are each monitored by dedicated stress responses, it is remarkable that no cell envelope stress response has been identified that monitors lipoprotein trafficking to the OM. It is notable that while the Rcs response is activated by lipoprotein trafficking defects caused either by LolB depletion or by inhibition of lipoprotein acylation, the consequences of Rcs activation are lethal to the cell (38, 39). In Δ*lpp* cells lacking the Rcs sensor lipoprotein (Δ*rcsF*) or response regulator (Δ*rcsB*), LolB depletion is well tolerated (38). Findings to date argue strongly that the Rcs system is not a response that relieves lipoprotein trafficking stress; rather, Rcs causes additional stress. Indeed, induction of Rcs during LolB depletion causes a lethal overproduction of the OM lipoprotein OsmB. In this study, we used genetic and chemical approaches to induce lipoprotein trafficking stress in E. coli, aiming to discover how the cell protects itself from such stress. We used a LolB depletion system to reduce Lol pathway trafficking efficiency, and we also made use of chemical inhibitors of LolCDE and Lsp. Our analysis identified the Cpx two-component system as a stress response that monitors lipoprotein trafficking. Cpx does this by detecting the OM-targeted lipoprotein NlpE while it transits the IM *en route* to the OM. Trafficking defects cause NlpE to accumulate in the IM and increase signaling to Cpx which mounts a protective response that preserves cell viability.

## RESULTS

### NlpE activates Cpx to protect against lipoprotein trafficking stress.

LolB is essential in wild-type E. coli, and such cells do not tolerate the stress caused by LolB depletion (Fig. 1B). This sensitivity to LolB depletion was shown to be due to two toxicities: the first caused by the abundant OM lipoprotein Lpp cross-linking inappropriately to cell wall peptidoglycan and the second resulting from hyperactivation of the Rcs stress response (38). The Cpx two-component system—consisting of the histidine kinase CpxA and the response regulator CpxR—was shown to be important for this tolerance (38). Indeed, mutations that activate CpxA enable deletion of *lolB* (38). However, Cpx and Rcs systems are known to cross-regulate each other (12, 40), so the physiological role of Cpx protection remained unclear in these engineered strains which lacked key components of the Rcs system (Δ*rcsB* or Δ*rcsF*) and did not produce the most abundant OM lipoprotein (Δ*lpp*).

We constructed a LolB depletion system in an E. coli *lpp*(ΔK58) Δ*osmB* background; these cells produce abundant Lpp (in a detoxified form, lacking the K58 residue that cross-links to peptidoglycan) and have an intact Rcs stress response (lacking only one regulon member, OsmB, that is lethally overproduced by Rcs during LolB depletion). Plasmid-borne LolB was expressed from an l-arabinose-dependent promoter, while the native *lolB* gene was deleted. Culturing the LolB depletion strain MG3487 without l-arabinose depletes cellular LolB levels, causing reduced lipoprotein trafficking and inducing stress. LolB depletion from *lpp*(ΔK58) Δ*osmB* cells was well tolerated (Fig. 1B). However, when *cpxR* was deleted from these cells, they became highly sensitive to LolB depletion, exhibiting significantly reduced viability (Fig. 1B). An OM lipoprotein, NlpE, is proposed to be a Cpx signaling molecule that activates the system when cells adhere to hydrophobic surfaces (41). NlpE is thought to transduce the adhesion signal from the OM to CpxA in the IM. We tested the involvement of NlpE for resisting lipoprotein trafficking stress and found that deleting *nlpE* caused *lpp*(ΔK58) Δ*osmB* cells to become highly sensitive to LolB depletion (Fig. 1B).

We wondered if lipoproteins other than NlpE were important in sensing trafficking stress. Prior work had systematically overproduced 90 of the 110 E. coli lipoproteins and tested their ability to activate the Cpx response (42). Only two Cpx-activating lipoproteins were identified, NlpE and YafY (an IM lipoprotein of unknown function) (42). We examined whether YafY contributed to protection against lipoprotein trafficking stress. Deleting *yafY* from the *lpp*(ΔK58) Δ*osmB* background had no effect on cell viability during LolB depletion (Fig. 1B). Hence, unlike NlpE, YafY played no role in combatting lipoprotein trafficking stress. Our findings suggest that the cell requires both NlpE and CpxR to combat lipoprotein trafficking stress caused by LolB depletion. The simplest model is that NlpE signals lipoprotein trafficking stress to CpxA, allowing CpxR to activate the stress regulon that protects the cell. Consistent with this model, loss of the IM sensor kinase CpxA causes the same severe sensitivity to LolB depletion as loss of NlpE or CpxR (see Fig. S1 in the supplemental material). NlpE appears to be the sole lipoprotein involved in Cpx activation in response to lipoprotein trafficking stress.

10.1128/mBio.00618-19.1 FIG S1 CpxA is required for tolerance of LolB depletion. Strains were tested for tolerance to LolB depletion. Expression of LolB was repressed by culturing in the absence of l-arabinose. Ten-fold serial dilutions of cultures are shown. Download FIG S1, TIF file, 0.6 MB. Copyright © 2019 May et al. 2019 May et al. This content is distributed under the terms of the Creative Commons Attribution 4.0 International license.

### The NlpE N-terminal domain is sufficient for resistance to lipoprotein trafficking stress.

**FIG 2:**
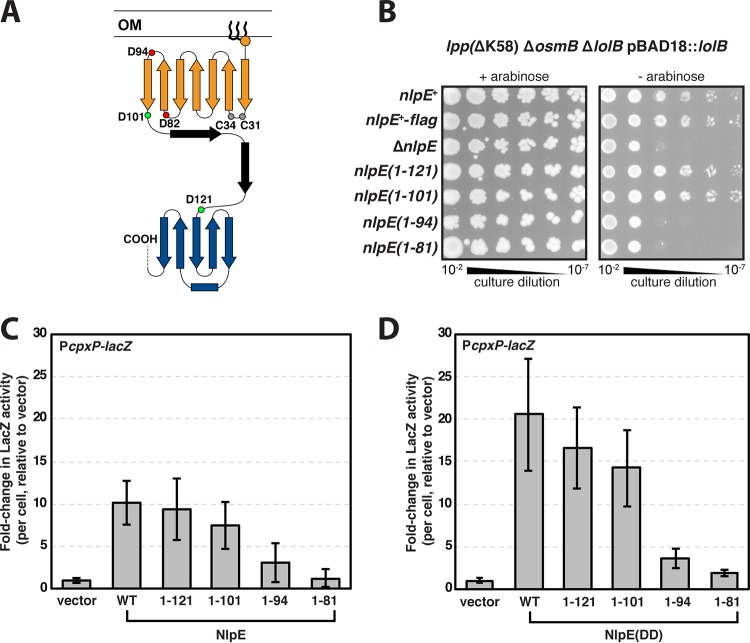
The NlpE N-terminal domain is sufficient for tolerance of LolB depletion and activation of Cpx. (A) Schematic of NlpE structure in its extended conformation. The N-terminal domain (orange) is joined to the C-terminal domain (blue) via a linker region (black). Sites of truncations are marked with spheres; green spheres indicate truncations that are able to activate Cpx, red spheres indicate truncations that fail to activate Cpx, and gray spheres show the Cys residues in a putatively redox-sensitive CXXC motif. (B) *nlpE* mutants were tested for their ability to tolerate LolB depletion (− arabinose) in an *lpp*(ΔK58) Δ*osmB* background. (C) Relative LacZ levels in Δ*nlpE* cells harboring a P*cpxP*-*lacZ* transcriptional reporter and overproducing plasmid-borne NlpE variants targeted to the OM. (D) Relative LacZ levels in Δ*nlpE* cells encoding a P*cpxP*-*lacZ* transcriptional reporter and overproducing plasmid-borne NlpE(DD) variants targeted to the IM. Data are means ± standard deviations.

E. coli NlpE is produced with two globular domains (43). The N-terminal domain, NlpE_1–101_, is homologous to bacterial lipocalin Blc (Fig. 2A). The C-terminal domain, NlpE_121–216_, contains an oligonucleotide/oligosaccharide-binding (OB) fold (Fig. 2A). The two domains are joined by a linker region (Fig. 2A). Prior work had solved the crystal structure of a domain-swapped E. coli NlpE dimer (43). This structure led to the prevailing model of NlpE signaling to Cpx: in its inactive (nonsignaling) form, NlpE is proposed to adopt a compact conformation mediated by interactions between N- and C-terminal domains (43). Adhesion cues were suggested to trigger an extended NlpE conformation that enabled it to span the periplasm, allowing the C-terminal domain to activate CpxA in the IM (43). This model implied that the NlpE C-terminal domain is the source of signaling.

Many Gram-negative species produce NlpE homologs that lack the C-terminal domain (43). The extended NlpE conformation model for signaling is inadequate to explain how these NlpE proteins function, since the N-terminal domain is small (approximately 50 Å) and unlikely to span the periplasm (>200 Å). This phylogenomic comparison suggested that the N-terminal domain may have a discrete signaling function. To test this hypothesis, we constructed strains that produce truncated NlpE proteins, each lacking the C-terminal domain (summarized in Table 1). We used CRISPR-Cas9 gene editing of the native *nlpE* gene to delete sequences encoding the C-terminal region. The resulting constructs were additionally tagged with FLAG epitopes at their C termini. The full-length *nlpE*^+^-*flag* construct exhibited wild-type activity and resulted in a toleration of LolB depletion (Fig. 2B). We found NlpE truncations that either removed the C-terminal domain (NlpE_1–121_) or removed both the C-terminal domain and the linker region (NlpE_1–101_) resulted in a toleration of LolB depletion (Fig. 2B). Despite the loss of the C-terminal domain, these truncated NlpE proteins were functionally equivalent to the full-length wild-type NlpE. Two larger truncations that included portions of the N-terminal domain (NlpE_1–94_ and NlpE_1–81_) were nonfunctional and resulted in cells that were as sensitive to LolB depletion as Δ*nlpE* cells (Fig. 2B). Therefore, our data demonstrate that the NlpE N-terminal domain is sufficient to overcome stress caused by LolB depletion.

**TABLE 1:** Summary of NlpE constructs in this study

Name	Description	Membrane targeting	Cpx activation
NlpE	Full-length wild-type NlpE	OM trafficked	Yes
NlpE(DD)	Full-length NlpE with N2D and N3D substitutions that cause avoidance of LolCDE	IM retained	Yes
NlpE_1–121_	NlpE that lacks the C-terminal domain	OM trafficked	Yes
NlpE(DD)_1–121_	Lacks the C-terminal domain; has the Lol avoidance signal	IM retained	Yes
NlpE_1–101_	Lacks the C-terminal domain and the linker region	OM trafficked	Yes
NlpE(DD)_1–-101_	Lacks the C-terminal domain and the linker region; has the Lol avoidance signal	IM retained	Yes
NlpE_1–94_	Lacks the C-terminal domain, the linker region, and a portion of the N-terminal domain	OM trafficked	No
NlpE(DD)_1–94_	Lacks the C-terminal domain, the linker region, and a portion of the N-terminal domain; has the Lol avoidance signal	IM retained	No
NlpE_1–82_	Lacks the C-terminal domain, the linker region, and a portion of the N-terminal domain	OM trafficked	No
NlpE(DD)_1–82_	Lacks the C-terminal domain, the linker region, and a portion of the N-terminal domain; has the Lol avoidance signal	IM retained	No
NlpE(C31S C34S)	Substitutions in N-terminal domain Cys residues proposed to form a disulfide bond	OM trafficked	Yes

### The NlpE N-terminal domain is sufficient to activate Cpx signaling.

Prior work established that NlpE overproduction activates the Cpx response (44–46). We assessed whether NlpE truncations that enable tolerance of LolB depletion could signal to activate Cpx. Each of the truncated *nlpE* alleles was cloned into pND18, a plasmid that allows l-arabinose-inducible expression of *nlpE* alleles and that has been used previously to demonstrate Cpx signaling (45). Plasmids were transformed into strain MG3593 that harbors a *cpxP*-*lacZ*^+^ transcriptional reporter whose expression is CpxR dependent and lacks the native *nlpE* gene. We supplemented the medium with l-arabinose to induce overproduction of plasmid-encoded NlpE constructs. LacZ levels in response to induction were measured. As expected, overproduction of full-length NlpE strongly activated the Cpx response (Fig. 2C). We measured equally strong Cpx activation when overproducing truncated NlpE proteins that either lacked the C-terminal domain (NlpE_1–121_) or lacked both the C-terminal domain and the linker region (NlpE_1–101_) (Fig. 2C). Truncations that included portions of the N-terminal domain were unable to activate Cpx (NlpE_1–94_ and NlpE_1–81_) (Fig. 2C). Importantly, Cpx activation by full-length NlpE or NlpE lacking the C-terminal domain was entirely dependent on the IM CpxA protein (see Fig. S2).

10.1128/mBio.00618-19.2 FIG S2 NlpE activation of Cpx requires the IM CpxA sensor kinase. Relative LacZ levels in Δ*nlpE* cells harboring a P*cpxP*-*lacZ* transcriptional reporter and overproducing plasmid-borne NlpE variants targeted to the OM or NlpE(DD) variants targeted to the IM. Data are means ± standard deviations. Increased basal Cpx signaling in Δ*cpxA*::*cam* strains is due to low level phosphorylation of the CpxR response regulator by acetyl phosphate (1). Download FIG S2, TIF file, 0.5 MB. Copyright © 2019 May et al. 2019 May et al. This content is distributed under the terms of the Creative Commons Attribution 4.0 International license.

Given that NlpE is an OM-targeted lipoprotein, we considered that truncations which fail to activate Cpx signaling may be too short to efficiently access CpxA but could remain otherwise functional for signaling. Therefore, we generated IM-localized NlpE constructs that could facilitate interaction with CpxA in the IM. We mutated the +2 and +3 residues in each of our *nlpE* alleles to Asp, generating the well-established strong IM retention signal that blocks lipoprotein entry into LolCDE (31). Indeed, the resulting IM localization of NlpE(DD) proteins was previously validated by cellular fractionation (42, 44). As in earlier experiments, we overproduced these constructs in MG3593 Δ*nlpE* and measured resultant LacZ levels. We detected stronger Cpx activation with IM-targeted full-length NlpE, in agreement with prior work (Fig. 2D) (42). The IM-localized NlpE(DD) proteins either lacking the C-terminal domain [NlpE(DD)_1–121_] or lacking both the C-terminal domain and the linker region [NlpE(DD)_1–101_] activated Cpx to the same extent as the full-length protein [NlpE(DD)] (Fig. 2D). The NlpE(DD) proteins that included truncations within the N-terminal domain did not activate Cpx [NlpE(DD)_1–94_ and NlpE(DD)_1–81_] (Fig. 2D). Clearly, the NlpE N-terminal domain is sufficient for Cpx activation; moreover, deletions within this domain abolish its ability to activate Cpx.

### The Cpx response protects against inhibitors of lipoprotein trafficking.

Since we had observed that NlpE activation of Cpx was important for resisting lipoprotein trafficking stress caused by LolB depletion, we sought to determine whether this effect was LolB specific or whether other lipoprotein trafficking stresses were also alleviated by NlpE and Cpx. To this end, we exploited two chemical inhibitors of lipoprotein trafficking to induce stress: we used compound 2 (Cpd2), a pyrazole compound that directly inhibits trafficking by interfering with LolCDE function (47), and we used globomycin (Glb), an inhibitor of signal peptidase II (Lsp) that indirectly blocks lipoprotein trafficking by preventing lipoprotein maturation (48–50).

We examined the effect of Δ*nlpE* and Δ*cpxR* mutations to E. coli survival when treated with Glb and Cpd2. Using a wild-type background, we observed Δ*cpxR* mutants were more sensitive to Glb and Cpd2 (Table 2). Inactivating *nlpE* caused cells to become more sensitive to Cpd2, but it did not appreciably affect sensitivity to Glb (Table 2). Both Glb and Cpd2 are efficiently effluxed from E. coli. CpxR regulates genes encoding efflux pumps that each work via the TolC OM efflux pore. Hence, one possibility was that Cpx protects against Glb and Cpd2 simply by increasing their efflux. We deleted *tolC* to inactivate all efflux systems and tested Glb and Cpd2 sensitivity in Δ*cpxR* and Δ*nlpE* derivatives. As expected, the Δ*tolC* mutation resulted in a striking increase in sensitivity to both compounds (Table 2). In this Δ*tolC* background, Δ*cpxR* mutants were more sensitive to both Glb and Cpd2 than Δ*tolC cpxR*^+^ cells. Clearly, CpxR is required to protect against the effects of Glb and Cpd2 in a manner that is independent of its regulation of efflux systems (Table 2). The Δ*tolC* Δ*nlpE* strain was more sensitive to Cpd2, but Glb sensitivity was unchanged compared to that in Δ*tolC nlpE*^+^ cells (Table 2), suggesting that while NlpE was important for resistance to Cpd2, it was not required for Glb resistance. Hence, NlpE is the sole activator of Cpx when LolCDE is inhibited by Cpd2, but NlpE is not absolutely required to trigger Cpx activation in response to Glb treatment. Our data show that the Cpx system is required to protect cells against diverse lipoprotein trafficking stresses, not just LolB depletion.

**TABLE 2:** MICs to lipoprotein trafficking inhibitors

Genotype	Glb (μM)	Cpd2 (μg/ml)
WT^*a*^	20	20
Δ*nlpE*	20	10
Δ*cpxR*	10	10
Δ*tolC*	0.63	0.31
Δ*tolC* Δ*nlpE*	0.63	0.16
Δ*tolC* Δ*cpxR*	0.31	0.16

a WT, wild type.

### Cpx senses lipoprotein trafficking stress by monitoring nascent NlpE transiting the IM.

As an OM-targeted lipoprotein, NlpE is hypothesized to signal from the OM. Structural data suggest that the folded N-terminal domain (NlpE_1–101_) is too small to span the periplasm to activate CpxA. However, our results demonstrate that NlpE_1–101_ is sufficient to activate Cpx signaling. We considered an alternate hypothesis for Cpx activation: NlpE signals lipoprotein stress to Cpx from the IM while it is being acylated and/or awaiting trafficking to the OM. Defects in either process would cause nascent NlpE to accumulate in the IM and correspondingly lead to increased Cpx activation.

**FIG 3:**
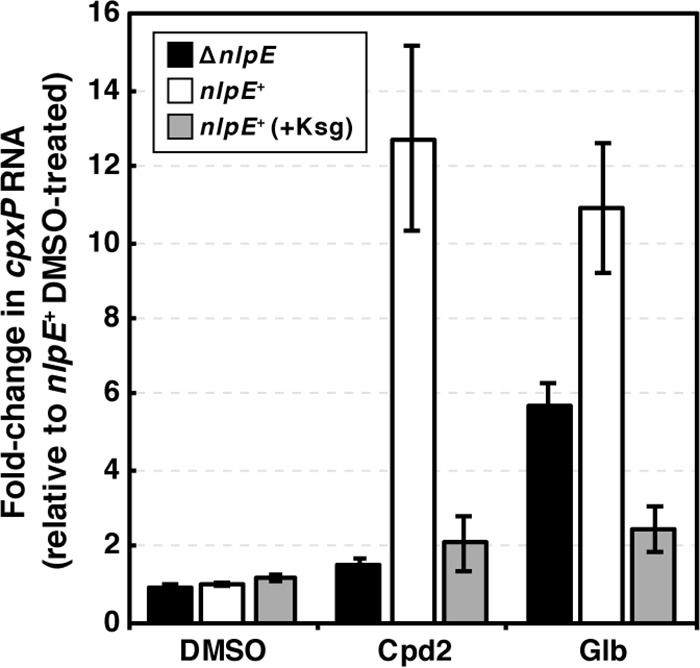
Inhibitors of lipoprotein biogenesis Glb and Cpd2 activate Cpx through NlpE. Cells were treated with either Glb or Cpd2 lipoprotein trafficking inhibitors (or DMSO vehicle control) for 20 min. RNA was then extracted and subjected to qRT-PCR to quantitate levels of *cpxP* mRNA. Ksg-treated cells (+Ksg) were treated with a sub-MIC of Ksg for 15 min prior to Glb or Cpd2 treatment (see Materials and Methods). Data are means ± standard errors of the means.

To test our hypothesis, we treated wild-type E. coli cells with sub-MIC amounts of Glb and Cpd2 and then examined the early Cpx response. We used reverse transcription-quantitative PCR (qRT-PCR) to measure the levels of *cpxP* mRNA following treatment (expression of *cpxP* is CpxR dependent) (51). We used dimethyl sulfoxide (DMSO) as a vehicle control for mock treatment. We detected a strong increase in *cpxP* levels in response to either Glb or Cpd2, indicating that the Cpx response was activated (Fig. 3). We hypothesized that NlpE is responsible for the Cpx activation in response to inhibitor treatment. Indeed, in a Δ*nlpE* strain, *cpxP* mRNA levels were not increased in response to Cpd2 treatment (Fig. 3). Cpx activation in response to Cpd2 is entirely dependent on NlpE signaling. This finding is consistent with the equivalent sensitivity of Δ*cpxR* and Δ*nlpE* mutants to Cpd2: the Cpx response is protective and it requires NlpE for activation. The Δ*nlpE* mutation only partially impaired Cpx activation following Glb treatment; we still detected increased *cpxP* transcription, albeit to a lesser extent (Fig. 3). These data showed that while NlpE does activate Cpx in response to stress caused by Glb, unknown additional factors also contribute to Cpx activation to Glb. This finding is also consistent with our MIC data: the Cpx response is protective, but Δ*nlpE* does not sensitize cells to Glb (like Δ*cpxR*) because Cpx can apparently be activated some other way. We have ruled out any contributions of Blc or YafY in responding to Glb (see Fig. S3).

10.1128/mBio.00618-19.3 FIG S3 The lipoprotein biogenesis inhibitor Glb activates Cpx partially through NlpE but not Blc or YafY. Cells were treated with Glb (or DMSO vehicle control) for 20 min. RNA was then extracted and subjected to qRT-PCR to quantitate levels of *cpxP* mRNA. Data are means ± standard errors of the means. Download FIG S3, TIF file, 0.4 MB. Copyright © 2019 May et al. 2019 May et al. This content is distributed under the terms of the Creative Commons Attribution 4.0 International license.

In a parallel set of Cpd2 and Glb experiments, we pretreated cells with the protein synthesis inhibitor kasugamycin (Ksg), which blocks the initiation of translation, stopping the production of new NlpE (and all other proteins) (52). During Ksg treatment, already-synthesized NlpE molecules have time to be secreted, acylated, and trafficked to the OM, but there are no new NlpE molecules going through these steps. Therefore, Ksg-treated cells that are subsequently treated with Glb or Cpd2 inform on signaling originating from OM-localized NlpE. This Ksg treatment approach was recently used to uncover OM-specific signaling in the Rcs stress system (53). Because Ksg-untreated cells continue to synthesize, acylate, and traffic new NlpE, while cells are treated with Glb and Cpd2, signaling can originate from OM-localized NlpE as well as from IM-localized NlpE that is *en route* to the OM. By measuring *cpxP* mRNA levels, we found that Ksg pretreatment blocked Cpx activation in response to both Cpd2 and Glb. To activate the Cpx response to these inhibitors, the cell must be synthesizing new proteins (Fig. 3). Since Ksg-treated cells do not activate the Cpx response to Cpd2 or Glb, NlpE signaling that activates Cpx in Ksg-untreated cells must originate from newly synthesized NlpE in the IM. It is clear that OM-localized NlpE molecules do not contribute to sensing lipoprotein trafficking stress caused by Cpd2 or Glb treatment.

### NlpE activates Cpx to protect against Cu toxicity.

**FIG 4:**
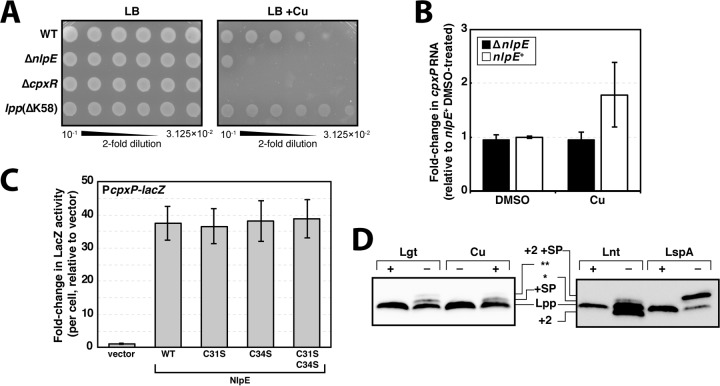
Cu impairs lipoprotein biogenesis and activates Cpx through NlpE. (A) Cultures were serially diluted on LB agar and LB agar supplemented with 4 mM CuCl_2_. (B) Cultures were grown in the presence of 3 mM CuSO_4_ to mid-log phase, and levels of *cpxP* mRNA were measured. Samples were prepared and analyzed together with samples presented in Fig. 3, the DMSO control presented is the same here as in Fig. 3 Data are means ± standard errors of the means. (C) Relative LacZ levels in Δ*nlpE* cells harboring a P*cpxP*-*lacZ* transcriptional reporter and overproducing plasmid-borne NlpE variants targeted to the OM. Data are means ± standard deviations. (D) Cultures were grown to mid-log phase in the presence (Cu +) or absence (Cu −) of 3 mM CuCl_2_. Lgt and Lnt replete (+) or deplete (−) samples were obtained by growing strains PAP9403 and KA472 in the presence or absence of arabinose; LspA activity was inhibited by treating cells with Glb (LspA −) in comparison to mock treatment (LspA +). Protein samples were taken and probed for Lpp by immunoblotting. Diacyl form Lpp is noted as +2. Lpp forms with signal peptides attached are noted as +SP. Peptidoglycan-bound Lpp forms are noted as * and **. See text for details.

Our findings indicated that NlpE is a sensor of lipoprotein trafficking stress that activates Cpx to protect the cell. Cpx was previously implicated in resistance to Cu toxicity (54, 55). We wanted to examine whether the ability of Cpx to sense stress caused by lipoprotein trafficking defects or Cu toxicity was due to distinct or linked functions. We also sought to determine whether NlpE was important for resisting Cu stress, since earlier studies yielded conflicting conclusions about its involvement. At first, *nlpE* (then named *cutF*) was found to be required for Cu resistance, since *nlpE* mutations sensitized cells to Cu (56). However, a later study demonstrated that while *cpxRA* mutants were sensitive to Cu, loss of *nlpE* had no effect on Cu resistance (55). Given these opposing findings, we reexamined Cu sensitivity in isogenic Δ*cpxR* and Δ*nlpE* mutants. We found that Δ*cpxR* caused a severe sensitivity to 4 mM Cu (Fig. 4A). The Δ*nlpE* mutation also sensitized cells to 4 mM Cu, though to a significantly lesser extent (Fig. 4A). Hence, both *cpxR* and *nlpE* genes are required to protect against Cu toxicity, though the loss of *cpxR* makes cells significantly more sensitive to Cu than loss of *nlpE*. The N-terminal domain of NlpE was sufficient to confer Cu resistance (see Fig. S4).

10.1128/mBio.00618-19.4 FIG S4 The NlpE N-terminal domain is sufficient to confer resistance to Cu. Cultures were serially diluted, plated on LB agar and LB agar supplemented with 4 mM CuCl_2_, and incubated overnight at 37°C. Download FIG S4, TIF file, 0.3 MB. Copyright © 2019 May et al. 2019 May et al. This content is distributed under the terms of the Creative Commons Attribution 4.0 International license.

Due to the clear involvement of both NlpE and CpxR in resistance to Cu, we would expect NlpE to activate Cpx when cells encounter Cu stress, as was previously reported. We used qRT-PCR to quantify levels of *cpxP* mRNA in wild-type E. coli and observed modest activation of Cpx within 20 min of Cu treatment (Fig. 4B), supporting earlier reports (55, 76). Importantly, we determined that this activation was entirely dependent on NlpE, since Δ*nlpE* cells failed to increase transcription of *cpxP* when treated with Cu (Fig. 4B).

Given that we detected NlpE-dependent activation of Cpx and that both factors are required for resistance to Cu, we sought to understand how NlpE senses Cu. Within the NlpE N-terminal domain there is a conserved 31-CXXC-34 motif that is in an oxidized state in the crystal structure, forming a disulfide bond (43). The chemically active Cys residues were hypothesized to offer a metal binding site in their reduced state (43). In nascent NlpE secreted from the cytosol, the Cys residues would be in a reduced state. The reaction of Cu with the thiol groups of these Cys residues would preclude disulfide bond formation, and this was proposed to change the structural properties of NlpE so that signaling to Cpx would occur (43). We tested this hypothesis directly by making cysteine to serine substitutions in the CXXC motif.

We used site-directed mutagenesis of an NlpE-encoding plasmid (pND18) to substitute each Cys to a Ser and to make a double Cys-to-Ser mutant. We transformed these plasmids into a *cpxP*-*lacZ* transcriptional reporter strain lacking the native *nlpE* gene (MG3593). NlpE mutant variants were each overproduced, and we assessed their ability to activate the Cpx response by measuring the levels of LacZ produced. We observed no effect of mutating either or both Cys residues; each of the mutants signaled as well as the wild-type protein (Fig. 4C). Clearly, the Cys residues in CXXC are not required for NlpE to activate Cpx, and altering the Cys residues is not sufficient to activate Cpx. Therefore, it is clear that the presence or absence of the C31-C34 disulfide bond is neither inhibitory nor stimulatory for NlpE signaling to Cpx.

### Cu inhibits lipoprotein maturation.

Since the CXXC motif was not required for NlpE signaling, we sought to understand how Cu treatment triggers NlpE to activate Cpx. We hypothesized that Cu could react with Cys residues in lipoproteins, impairing their chemical activity. Since the Cys^+1^ residues of lipoproteins are essential for their acylation, we thought that Cu may inhibit lipoprotein maturation and cause OM-targeted lipoproteins such as NlpE to remain in the IM (since precursors are unable to enter the LolCDE transporter). We assayed the abundant OM lipoprotein Lpp to test whether Cu treatment impairs lipoprotein maturation.

Cells were grown in the presence of 3 mM Cu, and Lpp was examined by SDS-PAGE and immunoblotting. Following Cu treatment, we observed two additional Lpp forms that migrated with higher apparent molecular masses in SDS-PAGE (Fig. 4D). We compared Lpp electrophoretic mobility following Cu treatment with Lpp from cells in which lipoprotein maturation was inhibited at different steps. We used Glb treatment to inhibit Lsp, and we used Lgt and Lnt depletion strains to limit the cellular activity of these essential enzymes (57, 58). Both Cu-treated and Lgt-depleted cells accumulated two additional Lpp forms that migrated slower than mature triacyl form Lpp (Fig. 4D). Lgt depletion prevents the *S*-diacylglyceryl modification at Cys^+1^ of Lpp and, as an indirect consequence, also prevents Lsp from processing the signal peptide, leading to pro-Lpp accumulation (Fig. 4D) (27). An additional species in Lgt-depleted samples that migrated more slowly than pro-Lpp was previously described as pro-Lpp that is linked to peptidoglycan fragments (marked * in Fig. 4D) (57). In contrast, Lsp inhibition by Glb leads to accumulation of diacylglyceryl-pro-Lpp (which migrates slower than pro-Lpp) (Fig. 4D), and depletion of Lnt caused an accumulation of diacyl form apo-Lpp (which migrated faster than triacyl form mature Lpp) and an additional peptidoglycan-linked species observed previously (marked ** in Fig. 4D) (59). Our comparative analysis suggests that Cu acts to block lipoprotein maturation. That Cu appears to mirror the effect of Lgt depletion is consistent with known Cu binding to Cys thiol groups (60). The Cys^+1^ of lipoproteins undergoes an Lgt-catalyzed thioester linkage to a diacylglyceride in the first step of maturation (see Fig. 1A). Our analysis supports a model in which Cu treatment blocks this Lgt reaction. By inhibiting maturation, OM-targeted lipoproteins, including NlpE, cannot be trafficked and remain in the IM (61). Our conclusion that Cu causes lipoprotein IM mislocalization is supported by the fact that an *lpp*(ΔK58) mutation confers resistance to Cu (Fig. 4A). Deleting *lpp* has long been known to confer Cu resistance (28, 62), but we show that deleting just the K58 residue provides Cu resistance. Lpp^K58^ is required for peptidoglycan cross-links. If Lpp is localized to the IM, cross-links between K58 and peptidoglycan are lethally toxic; deleting K58 detoxifies IM-localized Lpp and its retention in the IM is well tolerated. Since deleting K58 also prevents Cu toxicity, the simplest explanation is that one effect of Cu is to cause IM mislocalization of Lpp. Hence, in line with all our data, we propose that Cu causes NlpE to mislocalize in the IM and activate Cpx signaling in response to Cu stress.

## DISCUSSION

A stress response system that is dedicated to monitoring an OM biogenesis pathway must fulfil two key criteria: (i) it must be activated by the stress that results from defects in the pathway, and (ii) it must respond in a manner that protects the cell against the stress. It is firmly established that the σ^E^ and Rcs systems are stress responses dedicated to OMP and LPS biogenesis, respectively (9, 10, 14). Now, we identify the Cpx two-component system as a stress response that monitors lipoprotein trafficking to the OM. We have shown that Cpx is activated by several stresses at different steps in OM lipoprotein trafficking. Moreover, Cpx is required to protect cells against these stresses. Cpx is known to sense additional cell envelope stressors, including misfolded pilin subunits and disruption of periplasmic disulfide bonding (63, 64). Likewise, both Rcs and σ^E^ respond to additional noncanonical stressors in the cell envelope (17–19). A role for Cpx in monitoring OM lipoprotein trafficking is consistent with the current understanding that Cpx aims to protect the IM against stress (11, 65, 66). Defective OM lipoprotein trafficking is known to cause OM-targeted lipoproteins to mislocalize and accumulate in the IM where some are lethally toxic (38, 67). By protecting against defects in lipoprotein trafficking, Cpx is fulfilling its apparent mission to protect the IM. The mechanism by which Cpx protects against this stress awaits further exploration.

We have identified NlpE as the sensory component of the lipoprotein stress response. This is a newly identified function for NlpE, in addition to its surface adhesion sensory role from the OM. We have shown that Δ*nlpE* and Δ*cpxR* cells are similarly sensitized to diverse stressors affecting OM lipoprotein trafficking. It is important to note that lipoprotein trafficking is intimately linked to lipoprotein maturation, since only mature triacyl form lipoproteins are competent for trafficking. Hence, inhibitors of maturation cause trafficking stress by blocking trafficking and the correct localization of OM-targeted lipoproteins. NlpE is required to activate the Cpx response to trafficking stressors. However, NlpE is curiously only partially required to activate Cpx in response to Glb. Furthermore, while Δ*nlpE* cells are sensitized to Cpd2, LolB depletion, and copper, these cells are not sensitized to Glb. This apparent discrepancy may be due to the LspA-independent activity of Glb. Interactions between the lipid moiety of Glb and the IM bilayer could cause Cpx activation independent of the Glb effect on LspA and lipoprotein maturation. Notably, the LspA homolog in Mycobacterium tuberculosis is sensitive to Glb but is nonessential for cell viability (68). Yet, Glb remains mycobactericidal against M. tuberculosis mutants lacking *lspA*, implying other killing mechanisms exist (68). It is not known whether Glb has other cellular targets in E. coli. LspA-independent activity of Glb in E. coli has not been tested, but it is notable that no Glb-resistant mutations in LspA have yet been isolated. In contrast, E. coli mutants fully resistant to pyridineimidazoles such as Cpd2 map to either the LolC or LolE protein of the LolCDE transporter complex, demonstrating the highly specific activity of this drug.

NlpE was previously implicated in sensing cell adhesion to abiotic surfaces and subsequently activating Cpx (41). Although the structure of NlpE has been solved, the mechanism of this activation is unclear (43). Currently, it is suggested that cell adhesion elongates NlpE to allow the C-terminal domain to span the periplasm (43). This model has not been directly tested. In contrast to this model, we clearly show that the C-terminal domain is not required for sensing of lipoprotein trafficking stress by NlpE and Cpx. Indeed, our Ksg treatment data show that when protein synthesis is inhibited, the full-length NlpE that is already at the OM is unable to sense lipoprotein trafficking defects in response to Lol inhibition (with Cpd2). We show that the N-terminal domain is sufficient to sense lipoprotein trafficking stress. Moreover, Ksg treatment data show that the cell can only sense lipoprotein trafficking stress when it is synthesizing new NlpE. Newly synthesized NlpE is targeted for trafficking to the OM, and so defects in this process would cause its mislocalization and accumulation in the IM. The simplest model to explain how the cell senses lipoprotein trafficking stress is that Cpx monitors the presence of NlpE in the IM; trafficking defects lead to an accumulation of IM-mislocalized NlpE molecules and this accordingly increases Cpx activation to protect the cell. In this way, Cpx can gauge trafficking efficiency in the cell. The OM-localized NlpE is not informative as to the current state of lipoprotein trafficking. Our model of NlpE activating Cpx as it transits the IM is consistent with our finding that only the NlpE N-terminal domain is required. This small domain is unlikely to signal across the periplasm. Since this N-terminal domain is conserved in all NlpE homologs across diverse Gram-negative species, our data suggest that these diverse NlpE lipoproteins are competent for signaling. During the review of this study, another study of NlpE signaling was published that is consistent with our findings that the NlpE N-terminal domain signals to Cpx and that NlpE responds to lipoprotein trafficking stress (69).

The model lipoprotein trafficking stressors (LolB depletion and chemical inhibition) we have used are highly potent and likely represent the extreme end of the physiological spectrum. Nonetheless, they have been useful in identifying NlpE-Cpx as the lipoprotein trafficking stress response, and remarkably, this system is capable of protecting cells even against these extreme stressors. When might cells encounter conditions that cause stress in lipoprotein trafficking? One possibility is that environmental toxins such as Cu may impair trafficking. We have shown that one effect of Cu on the cell is to impede lipoprotein maturation (and hence trafficking). Both NlpE and Cpx were previously implicated in resistance to Cu stress, though it was unclear why they are involved (54–56). We have confirmed that Δ*nlpE* sensitizes cells to Cu stress, though Δ*cpxR* is comparatively more sensitive, and we showed that—at least in the early response—NlpE is required for Cpx activation to toxic Cu stress. There is strong underlying genetic evidence supporting the conclusion that Cu affects lipoprotein trafficking. Earlier screens had cataloged E. coli and *Salmonella* loci (*cutA-F*) that were important for resistance against Cu (56). Several of these can now be tied to lipoprotein biogenesis and trafficking. The *cutE* locus harbors *lnt* (28). The *cutF* locus encodes NlpE (56). The *cutC* locus was recently found to encode a small RNA, MicL, which acts to inhibit the production of the abundant OM lipoprotein Lpp (62). Cu sensitivity of *cutC* mutants was shown to be entirely due to inactivation of MicL (62). Indeed, cells lacking Lpp entirely (Δ*lpp*) are more resistant to Cu toxicity (62). By inhibiting lipoprotein maturation, Cu would cause IM mislocalization of OM-targeted lipoproteins. Previous work has demonstrated that at least two OM lipoproteins, OsmB and Lpp, are lethally toxic when allowed to mislocalize (38, 67). Several lines of evidence suggest that Cu is used by the innate immune system to help clear pathogens (70). Cu has many effects on the bacterial cell and several are well understood, we have shown that Cu additionally inhibits the maturation of lipoproteins. This effect of Cu would seem to have two consequences: it causes lipoprotein mislocalization and, because each of the OM assembly machines requires lipoproteins, it would damage the integrity of the OM permeability barrier.

## MATERIALS AND METHODS

### Bacterial strains, plasmids, and growth conditions.

Strains and plasmids used in this study are listed in Tables S1 and S2, respectively, in the supplemental material. Chromosomal mutant alleles were introduced by P1*vir* transduction. Null alleles were obtained from the Keio collection and their Kan^r^ cassettes were cured using pCP20, as required (71, 72). Spectinomycin-marked Δ*cpxR* and Δ*nlpE* alleles have been previously described (44, 45). Strains were grown in Lennox broth (LB) or agar at 37°C. LB was supplemented with ampicillin (Amp; 25 μg/ml), chloramphenicol (Cam; 20 μg/ml), kanamycin (Kan; 25 μg/ml), tetracycline (Tet; 25 μg/ml), spectinomycin (25 μg/ml), and l-arabinose (0.2% [wt/vol]) as required.

10.1128/mBio.00618-19.5 TABLE S1 Strains used in this study. Download Table S1, DOCX file, 0.1 MB. Copyright © 2019 May et al. 2019 May et al. This content is distributed under the terms of the Creative Commons Attribution 4.0 International license.

10.1128/mBio.00618-19.6 TABLE S2 Plasmids used in this study. Download Table S2, DOCX file, 0.1 MB. Copyright © 2019 May et al. 2019 May et al. This content is distributed under the terms of the Creative Commons Attribution 4.0 International license.

### Construction of truncated NlpE plasmids and chromosomal alleles.

Plasmid pND18 (45) encoding full-length NlpE was mutagenized using Q5 site-directed mutagenesis (NEB) with the oligonucleotides listed in Table S3 to generate plasmid derivatives encoding truncated NlpE. CRISPR-Cas9 editing was used to generate chromosomal *nlpE* truncations (73). An *nlpE* guide RNA was constructed in pCRISPR using primers CRISPR_NlpE_F and CRISPR_NlpE_R, generating plasmid pCRISPR*nlpE*. NlpE alleles were PCR amplified from plasmids using primers NlpE_ampli_F and NlpE_ampli_R, and the products were cotransformed with pCRISPR*nlpE* into the recombinogenic E. coli strain HME63 which carried *yafC*::Tn*10* markers (MG3670) (74). The resulting truncations of the native *nlpE* gene were confirmed by sequencing. The generated *nlpE* alleles were moved using colinkage with *yafC*::Tn*10*.

10.1128/mBio.00618-19.7 TABLE S3 Oligonucleotides used in this study. Download Table S3, DOCX file, 0.1 MB. Copyright © 2019 May et al. 2019 May et al. This content is distributed under the terms of the Creative Commons Attribution 4.0 International license.

### Efficiency of plating assays.

Efficiency of plating assays was used to determine the relative sensitivities of strains to CuCl_2_ or growth in the absence of l-arabinose (in strains with arabinose-dependent *lolB* expression). Assays were performed by preparing serial dilutions (2-fold or 10-fold) of overnight cultures (standardized by *A*_600_) in 96-well microtiter plates before replica plating onto LB agar and selective medium and then incubating plates overnight at 37°C.

### MIC determination.

Overnight cultures were diluted (10^5^ cells/ml) and transferred to a 96-well plate. Two-fold serial dilutions of either compound 2 or globomycin were prepared in DMSO and added to each of the cell suspensions. Plates were incubated statically, overnight at 37°C, and *A*_600_ was measured using a BioTek Synergy H1 plate reader. The MIC was determined as the lowest concentration that completely inhibited growth.

### β-Galactosidase assays.

LacZ levels were measured as previously described (75). Briefly, overnight cultures of strains carrying NlpE-encoding plasmids were subcultured 1:100 for 2 h in medium supplemented with 0.01% arabinose to induce NlpE production. An equivalent number of cells (as determined by *A*_600_) was collected and resuspended in 100 μl before being permeabilized with 30 μl 0.1% SDS and 40 μl chloroform. Z-buffer (830 μl) was added and *ortho*-nitrophenyl-β-d-galactopyranoside (ONPG) hydrolysis was measured every 1 min over a 15-min kinetic assay, allowing *V*_max_ to be calculated.

### Depletion of Lgt and Lnt.

To deplete Lgt or Lnt, strains harboring arabinose-dependent expression of Lgt or Lnt (PAP9403 and KA472, respectively) were grown overnight in LB supplemented with 0.2% l-arabinose. Strains were subcultured 1:100 in fresh LB broth without arabinose. Cultures were grown at 37°C for 2 h and then back diluted 1:50 in fresh LB broth and grown for 2 to 4 h to an *A*_600_ of ∼0.4 to 0.6. Control cultures grown in the presence of l-arabinose were grown for ∼2 h to an *A*_600_ of ∼0.4 to 0.6.

### Treatment with Glb and Cpd2.

Cultures of an arabinose-resistant MC4100 derivative (MG3178) were grown overnight and then subcultured 1:100 in fresh LB. Sublethal amounts of Cpd2 (0.25× MIC, 2.5 μg/ml), Glb (0.25× MIC, 2.5 μM), or CuCl_2_ (3 mM) were added. Subcultures were grown at 37°C to an *A*_600_ of ∼0.5 to 0.6 (∼2 h). Cell samples were taken and analyzed by immunoblotting.

### Whole-cell lysate preparation and immunoblotting.

Equivalent cell densities (normalized by *A*_600_) were pelleted by centrifugation (10,000 × *g* for 5 min). For Cu-treated cells, absorbance measurements were made at *A*_500_ to minimize interference by Cu. Cells were solubilized in Bugbuster (EMD Millipore) with added Benzonase and incubated at room temperature for 15 min. Samples were diluted 1:2 with 2× Tricine-SDS sample buffer (Novex) with 4% β-mercaptoethanol (BME). Lysates were incubated at 100°C for 5 min and then resolved on 16% Tricine gels (Novex) with Tricine-SDS sample buffer (Novex) at 125 V for 2.5 h. Resolved proteins were transferred to 0.2-μm nitrocellulose membranes which were then probed with polyclonal rabbit anti-Lpp (from the Silhavy laboratory collection of antiserum raised against denatured proteins, used at 1:800,000).

### qRT-PCR analysis.

Levels of *cpxP* mRNA were assayed as has been described with minor modification (53). Briefly, after a 15-min pretreatment with kasugamycin (Ksg) or a vehicle control (DMSO), mid-log-phase cultures of the strains were subsequently treated with 2% DMSO, 10 mg/liter Cpd2, 20 μM globomycin, or 4 mM CuSO_4_ for 20 min. RNA was prepared and qRT-PCR was performed using oligonucleotides listed in Table S3. Relative levels of RNA were calculated using the ΔΔ*C_T_* method relative to the DMSO-treated *nlpE^+^* sample and the actual efficiencies of the primer set. Data are the averages from three to four independent biological replicates ± the standard errors of the means (SEMs).

10.1128/mBio.00618-19.8 TEXT S1 Supplemental references. Download Text S1, DOCX file, 0.1 MB. Copyright © 2019 May et al. 2019 May et al. This content is distributed under the terms of the Creative Commons Attribution 4.0 International license.
