# Supplementary figures and images for: A Stress Response Monitoring Lipoprotein Trafficking to the Outer Membrane
Source: mBio. 2019 May 28;10(3):e00618-19. doi: 10.1128/mBio.00618-19 (PMC6538781; doi:10.1128/mBio.00618-19)

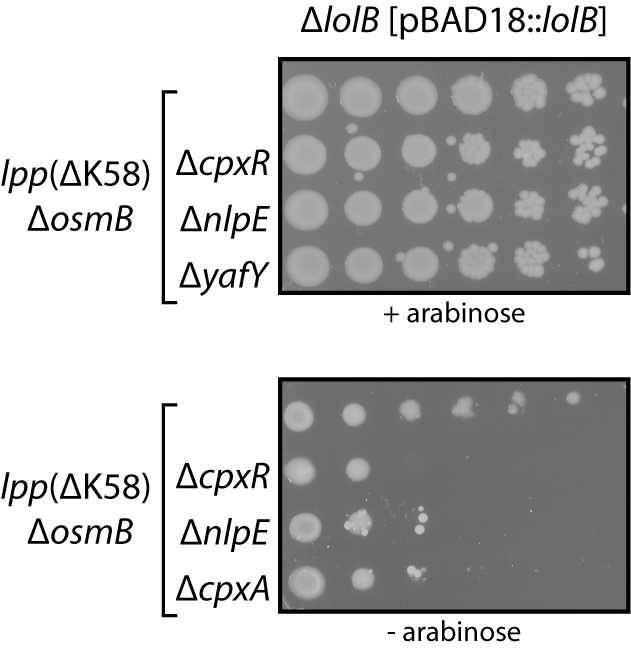

Supplement: FIG S1 [file mBio.00618-19-sf001.tif]

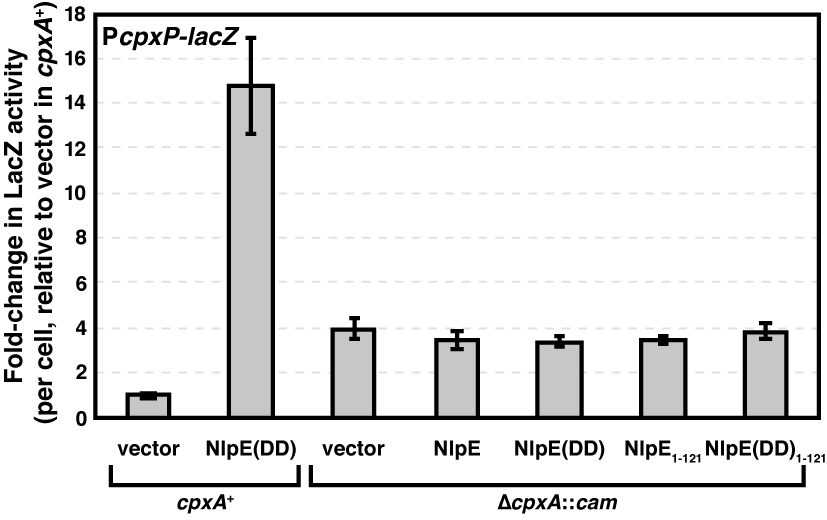

Supplement: FIG S2 [file mBio.00618-19-sf002.tif]

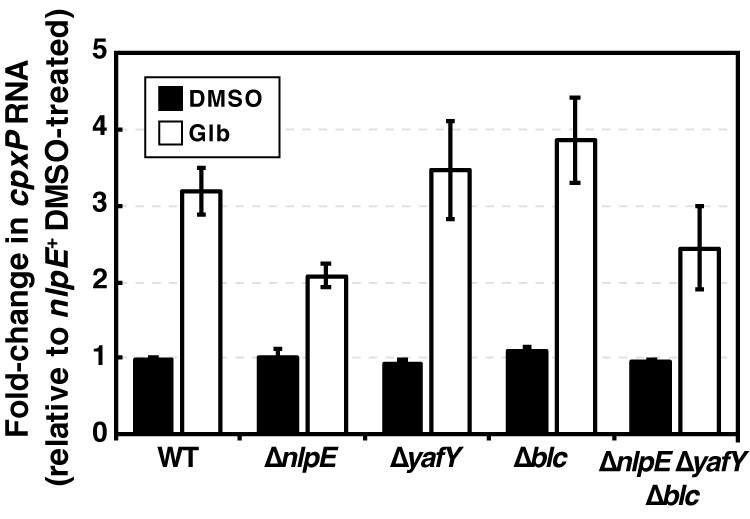

Supplement: FIG S3 [file mBio.00618-19-sf003.tif]

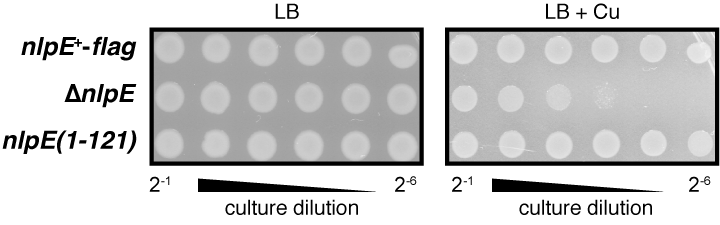

Supplement: FIG S4 [file mBio.00618-19-sf004.tif]
