# Supplementary material for: A Stress Response Monitoring Lipoprotein Trafficking to the Outer Membrane
Source: mBio. 2019 May 28;10(3):e00618-19. doi: 10.1128/mBio.00618-19 (PMC6538781; doi:10.1128/mBio.00618-19)
Supplement: TABLE S1 [file mBio.00618-19-st001.docx]

**Table S1: Strains used in this study**

| **Strain** | **Genotype** | **Reference** |
| --- | --- | --- |
| MC4100 | F^-^ *araD139* Δ(*arg-lac*)*U169 rpsL150 relA1 flbB5301 deoC1 ptsF25 thi* | (2) |
| KA472 | BW25113 *gut*::*kan*-*rrnB* TT-*araC*-P_BAD_-*lnt* Δ*lnt*::*spec* | (3) |
| PAP9403 | BW25113 Δ*lgt*::*kan* / pBAD18s-Cm-*lgt*-c-*myc*2 | (4) |
| MG3178 | MC4100 Ara^r^ | (5) |
| MG4004 | MG3178 *lpp*(ΔK58) *ynhG*::*kan* | This study |
| MG4005 | MG3178 Δ*nlpE::spec* | This study |
| MG4043 | MG3178 Δ*cpxR::spec* | This study |
| MG4082 | MG3178 Δ*yafY* | This study |
| MG4095 | MG3178 Δ*blc*::*kan* | This study |
| MG4096 | MG4003 Δ*yafY* Δ*blc*::*kan* | This study |
| MG4160 | MG3178 Δ*cpxA*::*cam* | This study |
|  |  |  |
| MG3179 | MG3178 Δ*tolC* | This study |
| MG4041 | MG3179 Δ*nlpE*::*spec* | This study |
| MG4042 | MG3179 Δ*cpxR*::*spec* | This study |
|  |  |  |
| HME63 | HME6 *mutS::bla* | (6) |
| MG3670 | HME63 *yafC*::Tn*10* / pCas9 | This study |
| **LolB depletion strains** | | |
| MG2162 | MG3178 Δ*lolB* / pBAD18::*lolB* | (7) |
| MG3487 | MG3178 *lpp*(ΔK58) *ynhG*::*kan* Δ*osmB* / pBAD18::*lolB* | This study |
| MG3737 | MG3487 Δ*cpxR::spec* | This study |
| MG3738 | MG3487 Δ*nlpE*::*spec* | This study |
| MG4051 | MG3487 Δ*yafY* | This study |
| MG3735 | MG3487 *yafC*::Tn*10* *nlpE(1-121)*-*flag* | This study |
| MG3768 | MG3487 *yafC*::Tn*10* *nlpE(1-101)*-*flag* | This study |
| MG3769 | MG3487 *yafC*::Tn*10* *nlpE(1-94)*-*flag* | This study |
| MG3770 | MG3487 *yafC*::Tn*10* *nlpE(1-81)*-*flag* | This study |
| MG3783 | MG3487 *yafC*::Tn*10* Δ*nlpE*::*spec* | This study |
| MG3820 | MG3487 *yafC*::Tn*10* *nlpE*^+^-*flag* | This study |
| **LacZ reporter strains** | | |
| MG3593 | NR754 λRS88[*cpxP'*-*lacZ*^+^] Δ*nlpE*::*spec* | This study |
| MG3609 | MG3593 / pBAD18 | This study |
| MG3948 | MG3593 / pND18 | This study |
| MG3949 | MG3593 / pND18(DD) | This study |
| MG3950 | MG3593 / pND18NlpE(1-121) | This study |
| MG3951 | MG3593 / pND18NlpE(DD)(1-121) | This study |
| MG3952 | MG3593 / pND18NlpE(1-101) | This study |
| MG3953 | MG3593 / pND18NlpE(DD)(1-191) | This study |
| MG3954 | MG3593 / pND18NlpE(1-94) | This study |
| MG3955 | MG3593 / pND18NlpE(DD)(1-94) | This study |
| MG3956 | MG3593 / pND18NlpE(1-81) | This study |
| MG3957 | MG3593 / pND18NlpE(DD)(1-81) | This study |
| MG3626 | MG3593 / pND18NlpE(C31S) | This study |
| MG4045 | MG3593 / pND18NlpE(C34S) | This study |
| MG4046 | MG3593 / pND18NlpE(C31S C34S) | This study |
|  |  |  |
| MG4161 | MG3593 Δ*cpxA*::*cam* / pBAD18 | This study |
| MG3648 | MG3593 Δ*cpxA*::*cam* / pND18 | This study |
| MG3649 | MG3593 Δ*cpxA*::*cam* / pND18(DD) | This study |
| MG3650 | MG3593 Δ*cpxA*::*cam* / pND18NlpE(1-121) | This study |
| MG3651 | MG3593 Δ*cpxA*::*cam* / pND18NlpE(DD)(1-121) | This study |
|  |  |  |
| MG3816 | NR754 λRS88 [*cpxP*-*lacZ*] *yafC*::Tn*10* *nlpE^+^-flag* | This study |
| MG3860 | NR754 λRS88 [*cpxP*-*lacZ*] *yafC*::Tn*10* Δ*nlpE*::*spec* | This study |
| MG3746 | NR754 λRS88 [*cpxP*-*lacZ*] *yafC*::Tn*10* *nlpE(1-121)-flag* | This study |
