# Supplementary material for: A Stress Response Monitoring Lipoprotein Trafficking to the Outer Membrane
Source: mBio. 2019 May 28;10(3):e00618-19. doi: 10.1128/mBio.00618-19 (PMC6538781; doi:10.1128/mBio.00618-19)
Supplement: TABLE S2 [file mBio.00618-19-st002.docx]

**Table S2: Plasmids used in this study**

| **Plasmid** | **Description** | **Reference/ Source** |
| --- | --- | --- |
| pBAD18 | Cloning vector for arabinose-inducible expression, Amp^R^ | (8) |
| pCas9 | Cas9 encoding plasmid, Cam^R^ | (9) |
| pCP20 | FLP recombinase plasmid, t.s. 30^o^C Cam^R^ Amp^R^ | (10) |
| pCRISPR | CRISPR locus encoding plasmid, Kan^R^ | (9) |
| pCRISPR*nlpE* | *nlpE* guide RNA cloned into CRISPR locus, Kan^R^ | This study |
| pND18 | NlpE cloned into pBAD18, Amp^R^ | (11) |
