# Supplementary material for: A Stress Response Monitoring Lipoprotein Trafficking to the Outer Membrane
Source: mBio. 2019 May 28;10(3):e00618-19. doi: 10.1128/mBio.00618-19 (PMC6538781; doi:10.1128/mBio.00618-19)
Supplement: TABLE S3 [file mBio.00618-19-st003.docx]

**Table S3: Oligonucleotides used in this study**

| **Name** | **Sequence (5’-3’)** | **Note** |
| --- | --- | --- |
| CRISPR_NlpE_F | aaacAATTTTACCCCAACCAGGATTGCAGTAGTTg | Cloning CRISPR guide *nlpE* RNA |
| CRISPR_NlpE_R | AAAACAACTACTGCAATCCTGGTTGGGGTAAAATT | Cloning CRISPR guide *nlpE* RNA |
| NlpE_ampli_F | CGGTCGGGAATAAAAAGAAGG | Amplify pND18 *nlpE* locus |
| NlpE_ampli_R | TATCCGGCCTACAAAAAACGTG | Amplify pND18 *nlpE* locus |
| NlpE_screen_F | GCATCGAAAGAGCGCAGG | Screen native *nlpE* locus |
| NlpE_screen_R | TAAACGCCTTATCCGGCCTAC | Screen native *nlpE* locus |
| CRISPR_NlpE_D1 | CGGGCGAAAGGCGATGCGCTGGAGATGCTCTAA CCCGTCTTGAGACAGAAACAAACGCAA | Amplify pND18 truncation |
| CRISPR_NlpE_D2 | CGGGCGAAAGGCGATGCGCTGGAGATGCTCTAA CCCGTCTTGAGACAGAAACAAACGCAA | Amplify pND18 truncation |
| CRISPR_NlpE_D3 | GGTGAAAAGTCATATTATCGGGCGAAAGGCTAA CCCGTCTTGAGACAGAAACAAACGCAA | Amplify pND18 truncation |
| CRISPR_NlpE_D4 | GCGCGAACCGCTGACAAGCTGGTATTAACCTAA CCCGTCTTGAGACAGAAACAAACGCAA | Amplify pND18 truncation |
| NlpE_C51S_F | CGATTGCGAAGGAATCGAAACCT | Construct C31S mutation |
| NlpE_C51S_R | GCAGACGGCAGCACGCCG | Construct C31S mutation |
| NlpE_C54S_F | CGATaGCGAAGGAATCGAAACCT | Construct C34S mutation |
| NlpE_C54S_R | CGGCGTGCTGCCGTcTGC | Construct C34S mutation |
| NlpE_DD_F | GTgatgatCGGGCCGAAGTCGATACG | Construct IM-retained NlpE |
| NlpE_DD_R | ATCCCATCAGAGTAAAGAGGCTGATTAC | Construct IM-retained NlpE |
| NlpE_FLAG_F | GACTACAAAGACGATGACGACAAGTAA CCCGTCTTGAGACAGAAACAAA | Cloning into pND18 |
| NlpE_del_142-236_R | GCTGGATTGTGCCGCTTCCA | Clone NlpE(1-121) into pND18 |
| NlpE_delta2 | GAGCATCTCCAGCGCATCG | Clone NlpE(1-101) into pND18 |
| NlpE_delta3 | GCCTTTCGCCCGATAATATGACT | Clone NlpE(1-94) into pND18 |
| NlpE_delta4 | GGTTAATACCAGCTTGTCAGCGGT | Clone NlpE(1-81) into pND18 |
| cpxP_F | TGCTGAAGTCGGTTCAGGCGATAA | qRT-PCR of *cpxP* |
| cpxP_R | TCTGCTGACGCTGATGTTCGGTTA | qRT-PCR of *cpxP* |
| ubiJ_F | GTTATCGCCTACGCCAGTGT | qRT-PCR of *ubiJ* |
| ubiJ_R | GGCTTTGCTGATTCCTTCAG | qRT-PCR of *ubiJ* |
