# Supplementary material for: A Stress Response Monitoring Lipoprotein Trafficking to the Outer Membrane
Source: mBio. 2019 May 28;10(3):e00618-19. doi: 10.1128/mBio.00618-19 (PMC6538781; doi:10.1128/mBio.00618-19)
Supplement: TEXT S1 [file mBio.00618-19-s0001.docx]

**SUPPLEMENTARY REFERENCES**

1. **Wolfe AJ**, **Parikh N**, **Lima BP**, **Zemaitaitis B**. 2008. Signal integration by the two-component signal transduction response regulator CpxR. J Bacteriol **190**:2314–2322.

2. **Casadaban MJ**. 1976. Transposition and fusion of the *lac* genes to selected promoters in *Escherichia coli* using bacteriophage lambda and Mu. J Mol Biol **104**:541–555.

3. **Armbruster KM**, **Meredith TC**. 2017. Identification of the Lyso-Form N-Acyl Intramolecular Transferase in Low-GC Firmicutes. J Bacteriol **199**.

4. **Pailler J**, **Aucher W**, **Pires M**, **Buddelmeijer N**. 2012. Phosphatidylglycerol::Prolipoprotein Diacylglyceryl Transferase (Lgt) of Escherichia coli Has Seven Transmembrane Segments, and Its Essential Residues Are Embedded in the Membrane. J Bacteriol **194**:2142–2151.

5. **Button JE**, **Silhavy TJ**, **Ruiz N**. 2007. A suppressor of cell death caused by the loss of σᴱ downregulates extracytoplasmic stress responses and outer membrane vesicle production in *Escherichia coli*. J Bacteriol **189**:1523–1530.

6. **Costantino N**, **Court DL**. 2003. Enhanced levels of lambda Red-mediated recombinants in mismatch repair mutants. **100**:15748–15753.

7. **Grabowicz M**, **Silhavy TJ**. 2017. Redefining the essential trafficking pathway for outer membrane lipoproteins. Proc Natl Acad Sci USA **114**:4769–4774.

8. **Guzman LM**, **Belin D**, **Carson MJ**, **Beckwith J**. 1995. Tight regulation, modulation, and high-level expression by vectors containing the arabinose PBAD promoter. J Bacteriol **177**:4121–4130.

9. **Jiang W**, **Bikard D**, **Cox D**, **Zhang F**, **Marraffini LA**. 2013. RNA-guided editing of bacterial genomes using CRISPR-Cas systems. Nat Biotechnol **31**:233–239.

10. **Datsenko KA**, **Wanner BL**. 2000. One-step inactivation of chromosomal genes in *Escherichia coli* K-12 using PCR products. Proc Natl Acad Sci USA **97**:6640–6645.

11. **Danese PN**, **Snyder WB**, **Cosma CL**, **Davis LJ**, **Silhavy TJ**. 1995. The Cpx two-component signal transduction pathway of *Escherichia coli* regulates transcription of the gene specifying the stress-inducible periplasmic protease, DegP. Genes Dev **9**:387–398.
